# Some Thoughts on Pellagra*Abstract of paper read before Section of State Medicine, Texas State Association, Galveston, May, 1916.

**Published:** 1916-09

**Authors:** I. L. Van Zandt

**Affiliations:** Fort Worth


### Some Thoughts on Pellagra.*

BY I. L. VAN ZANDT, M. D., FORT WORTH.

Controverting the idea that pellagra is due to an unbalanced ration, Dr. Van Zandt asks the advocates of this claim to answer these questions:

Why, while pellagra on the eastern hemisphere extends from Egypt to Poland, 200 miles further north than our Canadian border, in the western it is almost entirely limited to the Southern States?

Why the mitigating effects of the winter?

Why in this country for 410 years after the discovery of America there were no unbalanced rations and no pellagra, and yet in the next thirteen years these should have been developed by the thousands and tens of thousands?

He then reviews the history of pellagra on the eastern hemisphere. It was first noted by Casal in Spain in 1735. Twenty-five years later it appeared and developed rapidly in Italy. Later was found across the Alps in the Tyrol and other parts of Austria, then in most of the Balkan states and as far north as Poland. It also developed across the Mediterranean Sea in Egypt, Algeria and Tunis. In 1818 it was noted in the southwest corner of France, and from this point *spread* northward along the coast to the Garonne River, and up the left bank of that river, so that in a few years it had spread over the most of south France. About 1880 it began to decline, and now it has disappeared. He likens this to the spread of the yellow fever in the United States and the disappearance of malaria in a certain locality.

He calls attention to the fact that the outbreak of pellagra in the United States was in the first decade of this century, during which there was from southern Europe an immigration of 4,461,125 to be spread over the country, and asks was this a coincidence?

Traumatic cases, those following surgical operations, suggest that where the disease is prevailing, there are many unrecognized

*Abstract of paper read before Section of State Medicine, Texas State Association, Galveston, May, 1916.

cases. On this idea he suggests that Dr. Goldberger's feeding experiment in the Mississippi penitentiary would have been much more conclusive had it been carried out in New York or Montreal, where the disease was not prevailing.

The writer then suggests, as there are many unrecognized cases, the desirability of a Widal, Von Pirque, Wasserman or Abderholden test, so that these cases might have early treatment.

---

We have only two communications on pellagra in this issue. One is historical, and if Dr. Kerr is correct in the diagnosis he makes it is probable that others who were connected with Andersonville prison will come forward and add their evidence, some of which may be documentary.

Dr. Lee's letter, endorsing the theory held by Dr. Perdue, is very earnest.

I have just read a paper by Dr. I. L. Van Zandt of Fort Worth which is so sensible that I am publishing an abstract of same.

I have also had a letter from Dr. Patrick of Learned, Miss., in which he asks for the technique of intravenous administration of quinine.

The technique for this little operation is described at length in the paper by Dr. Wright of Monroe, La. The dihydrochloride of quinine is used. The powder is preferred as the solution sold in stock ampules seems to be irritating to the vein, but whenever the powder cannot be obtained or there is trouble in obtaining sterile salt solution, the ampules prepared for the purpose may be used. The administration is very simple and there is usually not the slightest irritation provided as much as 20 c.c. of the salt solution is used for each ten-grain dose. Dr. Wright advises a 20 c.c. all glass syringe and a 20-gauge needle.

No one can refuse to give quinine intravenously because he lacks conveniences. The quinine in powders already measured, or in solution in the ampules, may be carried in any buggy case, likewise a syringe and bottle of distilled water. The quantity used is so small that the distilled water does not have to be fresh, but must be sterile. Utensils may be found in the humblest home in which to boil the syringe and a smaller utensil in which to boil the distilled water or saline solution, which is preferable.

During the past month I have given quinine intravenously to several patients suffering from pellagra. All of these patients except one showed some improvement, but they were all also receiving other treatment. However, the improvement has been sufficiently marked to justify the use of quinine intravenously in other cases of pellagra. But I do not think that it will take the place of cacodylate of soda, soamin or salvarsan.

If the physician wishes to be on the safe side he can very easily follow all of the advocated treatments in each case. The diet, according to Dr. Goldberger; the alkali, according to Dr. Perdue; quinine, according to Dr. Dyer, or more preferably by the intravenous route, and some arsenical preparation, may all be used without either method interfering in any way with the other.

E. H. M.

---

Doctor, if you ever expect to ship any pathological specimens to Dr. Graham, State Bacteriologist, do not fail to read his paper in this issue, and we would advise you to file it for further use. Dr. Graham is a very conscientious, painstaking pathologist, and, if you prepare your specimens according to his direction, you may rest assured that you will receive the very best service it is possible for him to give you.

---

Your attention is called to the advertisement of Dr. Watson's sanitarium for the treatment of goiter and diseases of the ductless glands which appears in this issue of the JOURNAL. This sanitarium is the only one in the United States devoted exclusively to the treatment of goiter by non-operative measures. That the general profession welcomes and approves this method of treatment by quinine and urea is evidenced by the fact that this institution, opened in March of this year, has been compelled, already, to move into new quarters to accommodate the numerous patients.

If you are interested, write Dr. Leigh F. Watson, 419 Colcord Building, Oklahoma City, for further particulars. —ED.

---

Dr. F. S. White, of San Antonio, has purchased an interest in the Lakeside Sanitarium. It will be good news to Dr. White's friends and the public in general that they will again have the doctor's service in this capacity.

---

The forty-fourth annual meeting of the American Public Health Association will be held in Cincinnati, Ohio, October 24 to 27, 1916. General sessions will be held in Hotel Gibson, 413 Walnut Street.

---

Dr. J. M. Martin, of Dallas, is on an extended trip to New York, Chicago and Boston.

